# Recent advances in high-throughput approaches to dissect enhancer function

**DOI:** 10.12688/f1000research.11581.1

**Published:** 2017-06-19

**Authors:** David Santiago-Algarra, Lan T.M. Dao, Lydie Pradel, Alexandre España, Salvatore Spicuglia

**Affiliations:** 1 Aix-Marseille University, TAGC, Marseille, France

**Keywords:** gene transcription, enhancer function, MPRA, STARR-seq, CRISPR

## Abstract

The regulation of gene transcription in higher eukaryotes is accomplished through the involvement of transcription start site (TSS)-proximal (promoters) and -distal (enhancers) regulatory elements. It is now well acknowledged that enhancer elements play an essential role during development and cell differentiation, while genetic alterations in these elements are a major cause of human disease. Many strategies have been developed to identify and characterize enhancers. Here, we discuss recent advances in high-throughput approaches to assess enhancer activity, from the well-established massively parallel reporter assays to the recent clustered regularly interspaced short palindromic repeats (CRISPR)/Cas9-based technologies. We highlight how these approaches contribute toward a better understanding of enhancer function, eventually leading to the discovery of new types of regulatory sequences, and how the alteration of enhancers can affect transcriptional regulation.

## Introduction

Gene expression is precisely regulated by a combination of promoters and gene-distal regulatory regions, known as enhancers ^1, 2^. With the increasing awareness of the important role of enhancers in normal development as well as in disease, there is strong scientific interest in identifying and characterizing these elements. This is a challenging task because an enhancer does not have to be located directly adjacent to the gene it regulates. Putative enhancers can be identified across entire genomes based on open chromatin regions (e.g. based on DNase I-seq or ATAC-seq) or chromatin signatures (H3K4me1, H3K27ac), which map the potentially active enhancers ^3^. Although useful, these approaches do not provide direct proof of enhancer function, nor do they allow insights into the discrete sequences required for enhancer activity. Therefore, it is crucial to test whether genomic regions actually function as *bona fide* enhancers in living cells or tissues.

In recent years, various powerful techniques that incorporate high-throughput sequencing into reporter assays have enabled quantitative and straightforward measurements of enhancer activity of thousands of regulatory elements. More recently, the advent of clustered regularly interspaced short palindromic repeats (CRISPR)-related approaches allows massively assessing the relevance of enhancer function in the endogenous context. This review summarizes the assays developed for functional genome-wide testing of enhancer activity and their limitations as well as the main findings that have been gathered using these techniques.

## Principle of high-throughput reporter assays

**Figure 1.**
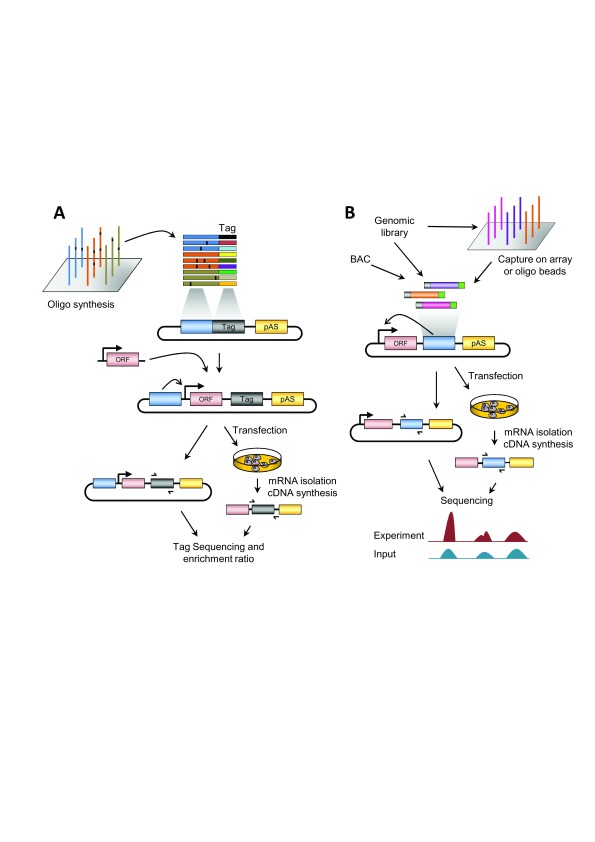
Principle of high-throughput assays for enhancer activity. ( **A**) Overview of massively parallel reporter assay (MPRA). The test sequences (wild-type, variants, etc.) are generally synthesized *in silico* by massive oligonucleotide synthesis with unique barcode tags and cloned into the plasmid backbone. Tags can be synthesized along with the test sequences or added after synthesis by polymerase chain reaction (PCR) amplification. A basal promoter and a reporter open reading frame (ORF) are inserted between the tested element and tag sequences. The reporter library is then transfected into cultured cells. Subsequently, mRNA is isolated and cDNA synthesized. The tags are sequenced before (plasmid library pool, for normalization) and after the transfection. The difference in the enrichment of each barcode is proportional to the enhancer activity of the test sequence. In the case of post-synthesis addition of barcodes, an additional sequencing step is required at the first cloning step. ( **B**) Overview of self-transcribing active regulatory region sequencing (STARR-Seq). A genomic or bacterial artificial chromosome (BAC) library is cloned in the reporter plasmid, downstream of the ORF and upstream of the polyadenylation site (pAS). Alternatively, the regions of interest might be enriched by a capture approach. The reporter library is transfected into cultured cells. Subsequently, mRNA is isolated and cDNA synthesized. The cloned regions are sequenced from the plasmid library pool (input) and the cDNA. Differences in the enrichment with respect to the input are proportional to the enhancer activity. In both panels, the effect of the enhancer on the basal promoter is indicated by an arrow.

Episomal reporter assays have been widely used to characterize putative regulatory regions. Several high-throughput strategies have been developed, permitting the simultaneous analysis of hundreds of thousands of reporter plasmids at once. These have been the focus of several comprehensive reviews (e.g. 4– 6). These methods can be either qualitative (usually based on cell sorting) or quantitative (based on RNA-seq) and designed to test enhancer or promoter activity. Here, we will focus on recent quantitative methods aiming to characterize enhancers. In particular, two approaches have been widely used in recent years ( Figure 1; Table 1): massively parallel reporter assay (MPRA) and self-transcribing active regulatory region sequencing (STARR-seq).

**Table 1.** Examples of high-throughput functional assays of mammalian enhancers.

Original technique	Specific Name	DNA origin	Application	Specific features	No. of regions ^1^	Size (bp)	Cell lines	Promoter	Species	Ref.
MPRA		Synthetic	Characterize putative enhancers and their active region	Centered on TF sites	2,104	145	K562, HepG2	SV40	Human	12
MPRA	CRE-seq	Synthetic	Characterize genomic regions with predicted function as enhancers	Mutation of TF binding sites	2,100	130	K562	Hsp68	Human	9
FIREWACh		Genomic	Identification of enhancers from restriction enzyme accessible regions	Isolation of GFP-positive regions	84,240	154	ESC	Minimal FpG5	Mouse	25
STARR-seq	CapSTARR-seq	Genomic	Identification of enhancers from DHS	Capture of DHS	7,542	330– 430	P5424, 3T3	SCP1	Mouse	22
STARR-seq		Genomic	Characterize genetic variants in regulatory elements from 95 subjects	Capture of DHS	104	402	HepG2	SCP1	Human	23
MPRA	CRE-seq	Genomic	Identification of regulatory elements	Capture of DHS	4,000	464	Retina	Minimal Rho	Mouse	24
MPRA	Sharpr-MPRA	Synthetic	Characterize nucleotides as activators or repressors in regulatory elements	Tiled oligonucleotide synthesis (30 or 5 bp resolution)	15,720	145	HepG2, K562	Minimal TATA, SV40	Human	13
MPRA		Synthetic	Identification of regulatory variants associated with eQTLs	Centered on eQTL variants	3,642	150	Lymphoblastoid, HepG2	Minimal TATA	Human	14
MPRA		Synthetic	Identification of regulatory variants associated with red blood cell traits	Three sliding windows with respect to the GWAS variants	2,756	145	K562, K562+GATA1	Minimal TATA	Human	15
STARR-seq	CapSTARR-seq	Genomic	Identification of promoter regions with enhancer activity	Capture of -200 to +50 bp with respect to the TSS of coding genes	20,719	250	K562, HeLa	SCP1	Human	33
MPRA	LentiMPRA	Synthetic	Identification of enhancers by chromosomal integration	Centered on several ChIP- seq peak signals	2,236	171	HepG2	pGL4.23 promoter	Human	26
CRISPR-Cas9		Synthetic	Identify endogenous enhancers bound by p53 and ERα TFs	Target p53 binding site in putative enhancers	685	N/A	BJ-RASg12v	N/A	Human	44

^1^Number of targeted DNA sequences, not necessarily the number of unique fragments that are tested

Bp, base pair; capSTARR-seq, capture-based self-transcribing active regulatory region sequencing; CHIP-seq, chromatin immunoprecipitation sequencing; CRE, *cis*-regulatory elements; CRISPR, clustered regularly interspaced short palindromic repeats; DHS, DNase I hypersensitive sites; eQTL, expression quantitative trait loci; ERα, estrogen receptor alpha; ESC, embryonic stem cell; FIREWACh, functional identification of regulatory elements within accessible chromatin; GFP, green fluorescent protein; GWAS, genome-wide association study; Hsp68, heat shock promoter 68; lentiMPRA, lentiviral massively parallel reporter assay; MPRA, massively parallel reporter assay; N/A, not applicable; Sharpr, systematic high-resolution activation and repression profiling with reporter-tiling; STARR-seq, self-transcribing active regulatory region sequencing; SCP1, super core promoter 1; SV40, simian virus 40; TF, transcription factor; TSS, transcription start site.

The MPRA method consists of the generation of a library of reporter constructs based on microarray synthesis of DNA sequences (generally, tested sequences are cloned upstream of a basal promoter) and unique sequence tags or barcodes (placed in the 3' UTR of the reporter gene). To increase the sensitivity and reproducibility, several barcodes could be added to any given sequence. The reporter library is then transfected into cell lines of interest and RNA sequencing of the barcodes is performed, thus providing a quantitative readout of the regulatory activity of the tested regions ( Figure 1A).

MPRAs have been used to investigate a number of biological questions. Initially, MPRA was designed to dissect the functional components of previously identified enhancers at single-nucleotide resolution ^7, 8^. Subsequently, a similar approach (also named CRE-seq) was used to functionally test ~2,000 genomic segments predicted by ENCODE to be enhancers, weak enhancers, or repressed elements ^9^ as well as test synthetic enhancers to model grammatical rules of regulatory sequences ^10, 11^. MPRA can be used to systematically assess the relevance of predicted regulatory motifs within enhancers. Kheradpour *et al.* tested ~2,000 predicted enhancers along with engineered enhancer variants containing targeted motif disruptions for key transcription factors (TFs) ^12^. In a follow-up study, Kellis’ lab developed a high-resolution MPRA approach (also named Sharpr-MPRA) that allowed genome-scale mapping of activating and repressive nucleotides in regulatory regions ^13^. Here, by synthesizing dense tiling of overlapping MPRA constructs, they managed to infer the regulatory effects of functional regulatory nucleotides with either activating or repressive properties ^11, 13^. Finally, MPRA can be used to test the impact of single nucleotide polymorphisms (SNPs) in order to identify functional regulatory variants linked to human traits or diseases. Two recent studies from the Broad Institute provide proof-of-concept for such approaches. Tewhey *et al.* used an improved version of the MPRA to analyze thousands of human expression quantitative trait loci (eQTL) to identify alleles that impact gene expression in lymphoblastoid cell lines ^14^. Ulirsch *et al*. used MPRA to test 2,756 variants linked to 75 genome-wide association studies (GWAS) loci involved in red blood cell traits ^15^. In both cases, CRISPR-mediated genetic engineering confirmed the relevance of the MPRA findings. Interestingly, some of the identified regulatory variants did not lie within known motifs, suggesting that they can influence DNA structure instead of binding of TFs ^15^.

A new innovative method named STARR-seq was introduced by Alexander Stark and colleagues ^16^. STARR-seq is an MPRA (reviewed in 17) aimed to identify and quantify transcriptional enhancers directly based on their activity in entire genomes ( Figure 1B). In brief, a bulk of DNA fragments from arbitrary sources is cloned downstream into the 3' UTR of a GFP reporter gene. Once in cellular context, active enhancers will activate the upstream promoter and transcribe themselves, resulting in reporter transcripts among cellular RNAs. Thus, each reporter transcript contains the reporter gene and the “barcode” of itself. These reporter transcripts can be isolated separately by targeted PCR and eventually detected by high-throughput sequencing. In this way, the activity of millions of putative enhancers can be measured simultaneously without being affected by the location of the candidate sequences and their orientation. The main advantage over the classical MPRA is that the tested sequence itself is used as a “barcode”, substantially simplifying the whole procedure to quantify enhancer activity. Stark’s lab used the STARR-seq approaches to ask several basic mechanistic questions of enhancer biology in *Drosophila*, including (i) identification and characterization of cell-type-specific ^16, 18^ and hormone-responsive enhancers ^19^, (ii) the impact of *cis*-regulatory sequence variation on enhancer activity and evolution ^20^, and (iii) dissecting the basis of enhancer core-promoter specificity ^21^.

STARR-seq has been applied to human cells by utilizing selected bacterial artificial chromosomes (BACs) ^16^; however, with the complexity and size of mammalian genomes, this technique is not easily implemented, making the formulation of representative libraries a challenge and a very high sequencing depth a necessity. To avoid this issue, we developed a capture-based approach (named CapSTARR-seq) to assess a subset of mouse DNase I hypersensitive sites (DHSs) found in developing thymocytes ^22^. Here, the regions of interest are captured using custom-designed microarrays and cloned into the STARR-seq vector, thus providing cost-effective and accurate quantification of enhancer activity in mammals. Similar approaches have been published by other labs, including capture of natural genomic variants ^23^ and test of DHSs from the central nervous system using a capture approach with oligo-baits ^24^. Alternatively, it could be possible to directly clone open chromatin regions, as described in the functional identification of regulatory elements within accessible chromatin (FIREWACh) method ^25^.

## Potential caveats of high-throughput reporter assays

The DNA sources used for testing are a potential issue of high-throughput reporter assays. Most MPRA approaches have used massive oligonucleotide synthesis ( Figure 1A), which allows the precise definition of tested regions as well as custom modifications of underlying sequences. However, there are currently two limitations to this approach. On the one hand, the size of the tested fragment is limited to ~200 bp (including the adaptors), which might prevent testing the full regulatory regions. On the other hand, there is a limitation in the number of oligonucleotides that can be synthesized (currently <100,000). These constraints are expected to be overcome in the near future with the improvement of oligonucleotide synthesis technologies.

In the STARR-seq approach, the DNA fragments are cloned within the transcribed region ( Figure 1B), which is very convenient because their sequences provide direct information about enhancer activity. However, it also introduces a source of potential artifacts, as some sequences might influence transcript stability instead of enhancing transcription. This potential bias could be avoided by comparing the results of tested regions on both orientations, allowing one to filter out the effects of strand-specific transcript-stabilizing effects.

A general concern about the episomal reporter assays is that they may not accurately reflect the function of enhancer elements in their endogenous context. To partially circumvent this caveat, chromatinized adeno-associated ^11, 24^ and lentiviral MPRAs ^25, 26^ have also been performed. These methods capacitate reporter assays within cells or tissues that are difficult to transfect. Certainly, an equally valid argument is that episomal reporter assays allow the unbiased study of enhancer function independently of any “perturbing” chromatin or genomic context. Interestingly, a recent study performed a systematic comparison of chromosomal versus episomal enhancer activity using integrative and non-integrative versions of a lentiviral-based reporter assay ^26^. Although the chromosomally based reporter assay was more predictable by epigenomic and sequence-based models, both reporter assays remained relatively well correlated. Another alternative approach is the introduction of reporter genes throughout the genome using transposition systems (e.g. 27– 29). Although these approaches do not directly assess enhancer activity, they allow one to infer the regulatory context of endogenous loci.

## Some relevant findings

One interest of high-throughput enhancer assays is the possibility to explore enhancer function without preconceived notions, thus potentially leading to new unforeseen findings. A common observation of several studies is that many predicted enhancer regions did not show reporter activity ^9, 12, 13, 22, 26^. For example, only 26% of predicted enhancers based on chromatin signatures in K562 cells displayed enhancer activity in the reporter assays performed in the same line ^9^, suggesting that, in addition to histone modifications, additional sequence specificity, such as TF-binding sites, are essential determinants of *cis*-regulatory activity. Indeed, the concentration of TF-binding sites or motifs is highly predictive of strong enhancer activity ^13, 22, 30^. Alternatively, this could also indicate that not all of the required sequences are present in the tested regions or that endogenous promoter contexts are essential to the enhancer activity. Finally, it is also plausible that some open chromatin regions, while contributing to transcriptional regulation, have enhancer-independent functions ^31^ or lack classical enhancer functions ^32^.

Perhaps the most intriguing finding of functional enhancer assays comes from the observation that many core promoter regions display enhancer activity ^11, 16, 21, 33^. The original definition of enhancers implies the ability to activate gene expression at a distance, while promoters entail the capability to induce local gene expression. However, this basic dichotomy of *cis*-regulatory elements has been challenged by broad similarities between promoters and enhancers, such as DNA sequence features, chromatin marks, Pol II recruitment, and bidirectional transcription ^34^. For instance, H3K4me3, a histone modification generally found at promoter regions, has been also associated with active enhancers ^35– 37^. Assessment of enhancer activity by CapSTARR-seq showed that strong transcription start site (TSS)-distal enhancers are indeed associated with H3K4me3 enrichment at the endogenous loci ^22^. Several studies have also suggested that some promoters might play enhancer functions ^34^. The extent of this type of promoter and whether it actually functions to regulate the expression of distal genes have remained elusive. Now, several independent studies based on massive reporter assays reported widespread enhancer activity from TSS-proximal regions. By applying STARR-seq, Zabidi *et al.* screened the whole fly genome with the use of different core promoters obtained from either ubiquitously expressed housekeeping genes or developmentally regulated and cell-type-specific genes ^21^. They found that promoters of housekeeping genes were mainly regulated by promoter-proximal enhancers, while promoters of developmental and cell-type-specific genes required distally located enhancers. Ernst *et al.* found that active enhancers were enriched in DNase I sites overlapping TSS in human cell lines ^13^. Nguyen *et al*. performed a functional comparison of a subset of promoters and enhancers in mouse neurons using an integrative MPRA approach ^11^. Interestingly, gene promoters generated similar enhancer activity as compared to distal regulatory regions. In a recent study, we found that 2–3% of all human core promoters displayed enhancer activity in a given cell line ^33^. Compared to classical promoters and distal enhancers, these TSS-overlapping enhancers displayed distinct genomic and epigenomic features and were associated with housekeeping and stress response genes. CRISPR genomic deletions demonstrated that several core promoters with enhancer activity in the reporter assay are indeed involved in *cis*-regulation of distal gene expression in their natural context, therefore functioning as *bona fide* enhancers. Furthermore, human genetic variation within this type of promoter was associated with a strong effect on distal gene expression. Concomitantly, another study, using comprehensive genetic manipulation of promoter regions, reported frequent distal *cis*-regulation by loci associated with promoters of lncRNAs and, to a lesser extent, coding genes ^32^. Finally, two recent studies performing screens of *cis*-regulatory elements by CRISPR/Cas9-based approaches (see below) have found that the expression of some genes is controlled by distal gene promoters ^38, 39^. Overall, these findings open up the intriguing possibility that developmental traits or disease-associated variants lying within a subset of promoters might directly impact on distal gene expression.

## CRISPR-based approaches to assess enhancer function

As discussed above, a potential limitation of reporter assays is that candidate enhancers are studied outside their endogenous genomic context, which is likely required for their *in vivo* function. The advent of CRISPR-based technologies now allows the circumvention of this caveat. Several studies have performed systematic dissection of individual enhancers using either TALEN ^40^ or CRISPR-mediated mutagenesis ^41^. In these studies, a tiling single guide RNA (sgRNA), or TALEN, library covering selected enhancers was designed to perform *in situ* saturating mutagenesis screens, pinpointing sequences with either positive or negative impact on enhancer function. The CRISPR approach was subsequently extended to assess enhancer function within large genomic regions surrounding key loci ^38, 39, 42, 43^ or to screen for enhancer elements involved in specific gene regulation pathways ^44^ ( Table 1).

CRISPR-mediated mutagenesis is limited by the fact that a high density of sgRNAs is required to saturate all possible regulatory elements and specific mutations are difficult to implement genome-wide. In some cases, there might also be a bias with respect to the regions that can be targeted by the designed sgRNAs or limitations owing to transfection efficiency in particular cell types. Alternatively, nuclease-deactivated Cas9 (dCas9) can be fused to activator or repressor domains to precisely modify gene expression from promoters and distal regulatory elements ^45^. Based on this property, repressor and activator domains fused with dCas9 combined with a pool of sgRNAs have been used for comprehensive CRISPR-inactivation (CRISPRi) and CRISPR-activation screens targeting DHSs of a gene of interest ^46^ or an entire locus ^47^. In the former study, a reporter gene introduced at the place of the target genes was used to monitor enhancer activity. In the latter study, the screening criteria were based on the growth advantage or disadvantage provided by the change in expression of the enhancer-associated gene, thus providing proof-of-concept for screening of functional regulatory regions genome-wide. A current limitation of these approaches is that the screening strategy might be based on phenotypic features (such as cell growth fitness, developmental markers, etc.) instead of directly assessing the expression levels of regulated genes. To overcome this limitation, a new powerful method combined CRISPRi and single-cell RNA-seq ^48^, enabling high-throughput interrogation of enhancers at single-cell resolution and directly linking enhancer function, and their combinations, with its target gene(s). Although these approaches have been used so far to scan restricted genomic areas, they will likely be implemented in true genome-wide screens of regulatory elements in the coming future.

## Concluding remarks

The implementation of high-throughput reporter assays and CRISPR-based screens allows the experimental validation of enhancer activity in different cell types and cellular contexts ( Table 1). These assays are now robust and sensitive enough to be widely used as part of the toolkit for researchers interested in gene regulation. These approaches also led to unpredicted discoveries, such as the role of core promoters as enhancer-like regulators. One main limitation of these approaches remains the fact that they do not provide direct information towards uncovering enhancer target genes. Therefore, the combination of enhancer assays with recently developed 3C-related methodologies, such as 4C-seq, Hi-C or Capture Hi-C ^49^, should greatly facilitate the assignment of discovered enhancers to their putative target genes. Finally, with the expected decrease in the cost of sequencing and oligo synthesis, it will be possible to systematically test the impact of regulatory variants in different diseases and developmental contexts.

## Abbreviations

CRISPR, clustered regularly interspaced short palindromic repeats; DHS, DNase I hypersensitive site; MPRA, massively parallel reporter assay; sgRNA, single guide RNA; STARR, self-transcribing active regulatory region; TF, transcription factor; TSS, transcription start site.
